# Diet and Nutritional Status of Polish Girls with Rett Syndrome—A Case-Control Study

**DOI:** 10.3390/nu15153334

**Published:** 2023-07-27

**Authors:** Aneta Czerwonogrodzka-Senczyna, Magdalena Milewska, Paweł Kwiecień, Krzysztof Szczałuba

**Affiliations:** 1 Department of Clinical Dietetics, Faculty of Health Sciences, Medical University of Warsaw, 01-445 Warsaw, Poland; magdalena.milewska@wum.edu.pl; 2 Polish Rett Syndrome Association, 62-500 Konin, Poland; p.kwiecien@rettsyndrome.pl; 3 Department of Medical Genetics, 1st Faculty of Medicine, Medical University of Warsaw, 01-445 Warsaw, Poland; krzysztof.szczaluba@wum.edu.pl

**Keywords:** Rett syndrome, nutritional status, dietary assessment, body composition

## Abstract

(1) Background: Rett syndrome may be considered a disease strongly associated with nutritional disorders that are likely to require special management strategies, extending beyond what is usually required for children with other developmental disorders. The aim of the study was to assess the nutritional status and diet of Polish girls with Rett syndrome. (2) Methods: Each patient (study group = 49, control group = 22) underwent anthropometric measurements, including body weight and height, waist, hip and arm circumference, and skinfold measurement. The assessment of the diet was based on the analysis of 7-day menus and the Food Frequency Questionnaire (FFQ-6). Data were analyzed using Statistica 13.3. (3) Results: The majority of the girls with Rett syndrome were deficient in weight and height, and consumed fewer calories, less protein, dietary fiber, calcium, and iron than the control group. They also drank less fluid. Soft products that were easy to chew and considered to be high in energy value were significantly more common in the menus. (4) Conclusions: Girls with Rett syndrome are characterized by weight deficiencies, poor growth that deteriorates with age, and are at risk of food shortages. Various nutritional intervention strategies should be explored to reduce and, if possible, prevent malnutrition and cachexia in such patients.

## 1. Introduction

Rett syndrome is a neurodevelopmental disorder caused predominantly by mutations in the MECP2 gene [1]. The classic form of the disease occurs only in female children [2]. The main features of the disease include a period of normal development, followed by regression, with the loss of communication skills and hand function, the development of hand stereotypies and impaired gait [3]. In addition, Rett syndrome is associated with epilepsy and scoliosis. Poor growth is also one of the symptoms accompanying this disease [4,5,6,7]. It is caused by a number of factors affecting food intake, including feeding difficulties (despite good appetite), oromotor dysfunctions and other gastrointestinal disorders, as well as factors probably determined by the genotype and being an integral part of the disease [8]. There are additional neurological complexities, such as apraxia, autonomic dysfunction, including hyperventilation, sleep pattern disorders, or the development of scoliosis, each of which may exert an effect on food intake and growth. Contrary to numerous other neurological disorders, Rett syndrome may be considered a disease strongly associated with nutritional status disorders that are likely to require special management strategies, extending beyond what is usually required for children with other developmental disorders [9]. Rett syndrome is rare (1/10,000–15,000 female live births) [10]. Most clinicians typically have very few patients, and there is limited literature on the nutritional management of the disease. Moreover, the anthropometric assessment of the nutritional status, the measurement of body height in particular, constitutes a problem in this group of patients because some of them are unable to stand on their own or have deformities of the spine [11]. The Polish Rett Syndrome Association (PRSA) currently unites 160 families who are raising a child with Rett syndrome. The PRSA is dedicated to promoting awareness of the disease among doctors, therapists and society. The Association is actively involved in the European Rett Syndrome Association (ERSA), the International Rett Syndrome Foundation (IRSF) and the Australian InterRett Project. Moreover, the goals of the PRSA include direct assistance to children and their caregivers and cooperation with the Ministry of Health [12]. The aim of the study was to assess the nutritional status and diet of Polish girls with Rett syndrome. 

## 2. Materials and Methods

The study included 49 girls with Rett syndrome (study group) aged 8.7 ± 4.9 years and 22 healthy girls (control group) aged 10.7 ± 3.5 years. All girls in the study group were the wards of the Polish Rett Syndrome Association [12]. Patients for the study (study group) were recruited in June and July 2021, and the study was conducted in the autumn (October–December) of the same year. Patients with clinically diagnosed Rett syndrome confirmed by molecular testing were qualified for the study. The study group accounted for about 30% of the entire population of girls with Rett syndrome in Poland. The control group was recruited in January 2022 and the tests were carried out in February–March 2022. Healthy girls without any medical history were qualified. The control group was only a background for comparisons, because there are no studies on the nutritional status and diet of Rett syndrome in Poland. The study was conducted at the Department of Clinical Dietetics of the Medical University of Warsaw. The results of the study are part of the project entitled *Comprehensive assessment of energy needs, nutritional status and diet of girls with Rett syndrome*. The databases were prepared in April–November 2022. The examination was non-invasive and safe for the patient. The inconvenience was related to the need to come to the university and undergo an examination lasting about an hour.

Just under 50% (*n* = 25) of our patients have one of the six most common mutations in MECP2. 

As most of the girls in our study were clinically stable and had reached the age of 3–4 years, the staging was 3, except for 3 out of 49 patients who, as judged by their young age, were defined as having stage 2 to 3 disease. 

Gastrointestinal symptoms found in our patients included constipation and gastroesophageal reflux. Although probably of central origin, we have also included respiratory abnormalities, as these may mechanistically cause bloating and thus lead to upper alimentary tract disturbance. Twelve of 49 girls (24%) reported constipation, 11 (22%) had reflux disease, and 39% (*n* = 19) had respiratory abnormalities. The overlap of at least two of these features occurred in 15 girls (30%) in our cohort. Only one girl was PEG-fed.

In total, 14 out of 25 patients with one of the six common MECP2 mutations had one of the above gastrointestinal features.

### 2.1. Anthropometric Measurements

Each patient underwent anthropometric measurements, including body weight and height, waist, hip and arm circumference, and skinfold measurements: supra-iliac skinfold (SI), triceps skinfold (T) and subscapular skinfold (SS). Body weight was measured using the Radwag WPT 60/150 scales, with an accuracy of 100 g. If the patient could not stand unassisted on the scales, the measurement was performed together with the carer holding her, correcting the measurement for her body weight. The patient was weighed in light underwear. Body height was measured in the study group using an anthropometer with the patient in a lying position. The following measurements were taken: from the top of the head to the iliac spine, from the iliac spine to the knee joint space and from the knee joint space to the edge of the foot with an accuracy of 1 mm [13]. In girls who could stand unassisted and in the control group, the height was measured in a standing position with the use of a stadiometer with an accuracy of 1 mm. The circumferences were measured with a tailor’s tape, and the skinfolds were measured with the use of the Harpenden caliper with an accuracy of 1 mm. Waist circumference was measured at the location of the smallest circumference of the trunk, between the lower edge of the lowest rib and the upper edge of the iliac ala. The circumference of the hips was measured at the site of the largest circumference of the buttocks below the iliac alae. Skinfolds were measured on the non-dominant side of the body, three times, calculating the mean. The circumference of the arm was measured at midarm (between the acromion and the condyle of humerus) with an accuracy of 1 mm.

The following indices were calculated based on the measurements: BMI (body weight (kg)/body height (m)^2^), BMI z-score (the LMS method for WHO growth charts, and for children over 6.5 years of age the percentile for BMI was calculated using the calculator on the website: http://olaf.czd.pl/index.php?option=com_content&view=article&id=103:kalkulator (accessed on 16 January 2013); the BMI percentile was assigned the z-score value according to the publication by Kułaga et al. [14]), WHR (waist circumference (cm)/hip circumference [cm]), WHtR (waist circumference (cm)/body height (cm)), muscle mass (arm circumference − (T × 0.314)), %FAT (the Slaughter formula: if the sum of skinfolds ≤ 35 mm, then: 1.33 × (T + SS) − 0.13 × (T + SS)^2^ + 2.5) [15]. The interpretation of the BMI z-score was performed using the WHO growth charts (up to 5 years old) and national growth charts from the OLA and OLAF programs: significantly underweight: <(−)2 SD; underweight: <(−)1 SD–(−)2 SD; normal: (−)1 SD–(+)1 SD; overweight: >(+)1 SD–(+)2 SD; obese: >(+)2 SD and extremely obese: >(+)3 SD [16], and for girls > 18 years according to adult reference ranges [17]. Due to the occurrence of epilepsy, it was not possible to assess body composition using the method of electrical bioimpedance (Bio Scan 920-2 Maltron Int., Rayleigh, UK) in all patients. These measurements were performed in the control group and in only 7 girls with Rett syndrome.

### 2.2. Assessment of Nutrition

The assessment of the diet was based on the average intake for 7 days, comprising weekend days and the vitamin and mineral supplementation used, as well as oral nutrition support (ONS). The data from the menus were verified by a qualified dietitian who clarified and supplemented them during the interview. The FFQ-6 questionnaire was also analyzed to assess the frequency of consumption of products over 12 months. The information obtained from the FFQ-6 was interpreted by converting the original food frequency categories into semi-quantitative data that logically reflect the increasing intensity of the trait using their ranking, i.e., assigning the food frequency categories to conventional integers and converting them into real numbers, and expressing the food consumption frequency as times/day [18] (Table 1).

The data from the menus were evaluated using Dieta 6.0 program (developed by the National Institute of Public Health PZH-PIB in Poland). The obtained data were compared to nutritional reference values for the Polish population [19].

### 2.3. Statistical Methods

Descriptive statistics were performed using Statistica 13.3. Since most of the analyzed variables did not have a normal distribution (verified with the Lilliefors and Shapiro-Wilk tests), the data were presented as the mean, standard deviation (SD), median, quartiles and interquartile range (IQR). Differences in individual intergroup parameters were calculated using the Mann–Whitney U test, and the correlations between selected anthropometric variables with the Spearman’s rank correlation. The level of statistical significance was assumed at *p* < 0.05.

### 2.4. Approval of the Bioethical Committee

The study was approved by the Medical University of Warsaw Bioethical Committee (approval number KB/145/2021). Informed consent was obtained from all subjects involved in the study.

## 3. Results

### 3.1. Assessment of Nutritional Status

The mean Body Mass Index was 16.7 ± 6.9 kg/m^2^ in the study group, and 17.7 ± 3.4 kg/m^2^ in the control group (*p* = 0.15). BMI z-score was lower in the study group than in the control group: −0.8 ± 2.2 vs. −0.12 ± 1.09, respectively, with *p* = 0.07 (Table 2). The study group was more commonly characterized by body weight deficiency (*n* = 24, 48.9%) than the control group (*n* = 3, 13.6%), and excessive body weight (*n* = 7, 14.3% vs. *n* = 3, 13.6%, respectively), including extreme obesity (*n* = 2, 4.1% vs. *n* = 0.0%, respectively) (Table 3). Girls in the control group had significantly higher mean circumferences of the waist (63.4 ± 14.9 cm vs. 56.3 ± 10.5 cm, *p* = 0.03), hip (77.4 ± 12.0 cm vs. 63.3 ± 13.8 cm, *p* < 0.001) and arm (21.7 ± 3.9 cm vs. 17.3 ± 4.1, *p* < 0.001), as well as significantly higher muscle mass (17.2 ± 3.0 cm vs. 13.7 ± 2.9, *p* < 0.001) and body fat content calculated according to the Slaughter formula (25.6 ± 5.7% vs. 22.9 ± 6.7%, *p* = 0.93). Differences in %FAT were not reflected in the measurements with the electric bioimpedance method (19.7 ± 6.8% vs. 21.2 ± 13.8%, *p* = 0.89). Fat content >19% was found in 91% (*n* = 20) of control group girls and in 30% (*n* = 30) of girls with Rett syndrome, while low fat content (<15%) was confirmed in 10.2% (*n* = 5) of girls with Rett syndrome and in no control group girl. Both WHR (0.9 ± 0.1 vs. 0.8 ± 0.2, *p* < 0.001) and WHtR (0.5 ± 0.1 vs. 0.4 ± 0.1, *p* = 0.01) were significantly higher in the study group than in the control group (Table 2). The WHtR index ≥0.5, which indicates visceral obesity, was found in 38.8% (*n* = 19) of girls in the study group and in 9.1% (*n* = 2) of girls in the control group.

### 3.2. Assessment of Nutrition

The diets of girls with Rett syndrome significantly more commonly included soft products perceived as providing a high energy value, i.e., bananas (average times/day: 0.49 vs. 0.26, *p* = 0.01), potatoes (0.56 vs. 0.38, *p* = 0.04) or root vegetables in soups (0.48 vs. 0.34, *p* = 0.04), as well as products providing a large amount of protein, i.e., cold cuts (0.10 vs. 0.08, *p* = 0.06), cottage cheese (0.12 vs. 0.04, *p* = 0.09) or eggs (0.42 vs. 0.26, *p* = 0.07). Girls in the control group were more likely to consume sweets, i.e., chocolate (0.39 vs. 0.10, *p* < 0.001) and non-chocolate candies (0.15 vs. 0.03, *p* < 0.001), biscuits and cookies (0.23 vs. 0.14, *p* = 0.01), ice cream and pudding (0.21 vs. 0.07, *p* < 0.001) and salty snacks (0.18 vs. 0.09, *p* < 0.001), as well as cocoa (0.18 vs. 0.07, *p* = 0.07), kiwi and citrus fruits (0.43 vs. 0.21, *p* = 0.02), vegetables such as cucumber (0.51 vs. 0.34, *p* = 0.04), olives (0.14 vs. 0.03, *p* = 0.02), nuts (0.14 vs. 0.05, *p* < 0.001) and fruit juices (0.45 vs. 0.28, *p* = 0.08) and sweetened beverages (0.04 vs. 0.03, *p* = 0.00) (Table 4).

### 3.3. Dietary Intake

The diets of girls with Rett syndrome were characterized by a significantly lower energy value (median: 1248.5 kcal/d vs. 1683.9 kcal/d, *p* = 0.04) and carbohydrate content (181.6 g/d vs. 257.0 g/d, *p* < 0.001), including: starch (72.1 g/d vs. 132.3 g/d, *p* < 0.001), sucrose (39.2 g/d vs. 52.4 g/d, *p* = 0.04) and dietary fiber (11.7 g/d vs. 16.9 g/d, *p* = 0.00), compared to those in the control group. The percentage of people implementing the reference values for energy consumption in both groups was: 26.5% (*n* = 13) and 31.8% (*n* = 7), respectively (Table 5 and Table 6). However, the average implementation of reference values for certain minerals and vitamins was significantly higher in the study group. This included in particular: potassium (%AI: 124.2 vs. 90.6, *p* = 0.03) and zinc (%RDA: 120.4 vs. 84.0, *p* = 0.01), vitamins: B_1_ (%RDA: 111.1 vs. 81.4, *p* = 0.00), B_2_ (%RDA: 173.5 vs. 127.8, *p* = 0.01), B_6_ (%RDA: 170.5 vs. 122.2, *p* = 0.01), B_12_ (%RDA: 144.9 vs. 114.9, *p* = 0.02), folates (%RDA: 76.9 vs. 63.3, *p* = 0.04) and D (%RDA: 186.8 vs. 12.4, *p* < 0.001. When the daily intake of nutrients was converted into kg of body weight (kg b.w), it was found that girls with Rett syndrome consumed statistically significantly more of each of the analyzed macro and micronutrients, including sodium, saturated fatty acids, sucrose, cholesterol, dietary fiber and water (for example: energy—63.3 kcal/kg b.w vs. 46.4 kcal/kg b.w, *p* < 0.001, protein—2.5 g/kg b.w vs. 1.7 g/kg b.w, *p* < 0.001, fat—2.0 g/kg b.w vs. 1.2 g/kg b.w, *p* < 0.001, carbohydrates—8.9 g/kg b.w vs. 6.9 g/kg b.w, *p* = 0.017, sodium—107.5 mg/kg b.w vs. 79.1 mg/kg b.w, *p* < 0.001, SFA—0.9 g/kg b.w vs. 0.5 g/kg b.w, *p* < 0.001, sucrose—2.2 g/kg b.w vs. 1.2 g/kg b.w, *p* = 0.02, cholesterol—9.6 mg/kg b.w vs. 5.1 mg/kg b.w, *p* < 0.001, dietary fiber—0.6 g/kg b.w vs. 0.4 g/kg b.w, *p* = 0.01, water—62.4 mL/kg b.w vs. 44.6 mL/kg b.w, *p* < 0.001) (Table 5). Moreover, the percentage of individuals whose diets were compliant with the recommendations for the above mentioned ingredients was higher in the study group (Table 6) and this was mainly due to the increased use of supplements of those vitamins and ONS. The diets of control group girls contained higher amounts of polyunsaturated fatty acids (6.4 g/d vs. 4.3 g/d, *p* = 0.00), mainly including the n-6 family (5.5 g/d vs. 3.3 g/d, *p* = 0.00). Despite the significantly lower energy value of the diet, the percentage of proteins and fats in it was significantly higher in the diets of girls with Rett syndrome than in the control group: %E from protein (16.6 vs. 15.6, *p* = 0.02), %E from fats (28.4 vs. 24.7, *p* = 0.01) (Table 5). A low percentage of girls in the study group received a suitable amount of fluids (18.4% vs. 31.8%) (Table 6).

The existence of significant correlations between BMI z-score, arm circumference, muscle mass and other nutritional status indices was demonstrated in both the study and control groups (Table 7 and Table 8). Such correlations were not observed with regard to dietary intake.

**Figure 1:**
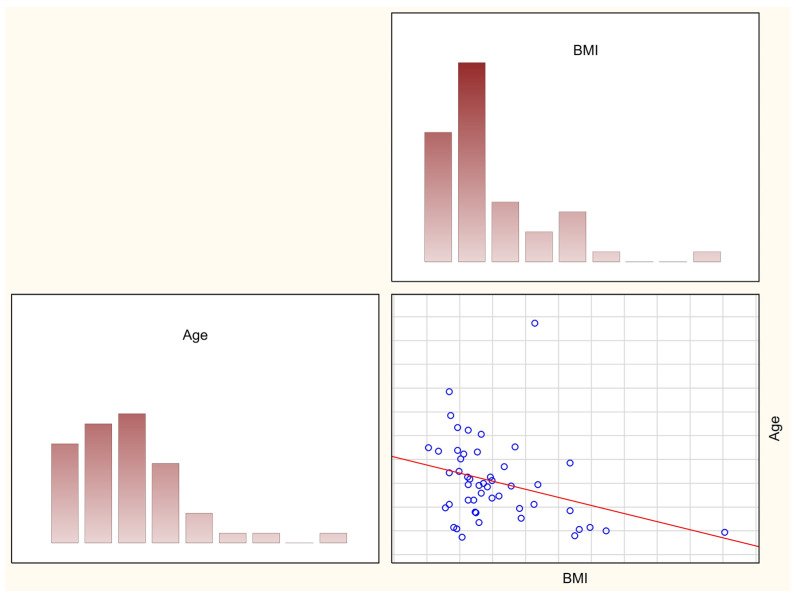
Correlation between body mass index and age in the study group (r = −0.36, *p* = 0.01).

In the study group a significant, negative correlation was also observed between age and body mass index (r = −0.36, *p* = 0.01) (Figure 1).

## 4. Discussion

The majority of the anthropometric parameters of girls with Rett syndrome were significantly lower than in the control group, which particularly applied to weight and height. Measuring body weight in this type of condition is considered a critical part of clinical evaluation because measuring body height is more problematic, especially since some girls or women are unable to stand on their own or had spinal deformities, while the mere comparison of body weight cannot be concluded without reference to body height, poor height gain and a comparison with a healthy population, also in the studies of other authors, seem to confirm significant deficiencies in the group of girls with Rett syndrome [11]. Few studies are available on the management of poor height gains in Rett syndrome, but they confirm the existing problem and the deterioration of parameters with age [6,7,20,21,22]. Although two people with extreme obesity were identified in the group of Polish girls with Rett syndrome, significantly more patients had BMI below the reference ranges for age and sex (48.9% vs. 13.6%). Similarly to the results published by other authors, the median BMI z-score was also lower than in the control group and BMI significantly decreased with age [7,20,21]. The analyses performed only compare individual variables between groups, and the influence of other factors cannot be completely excluded.

Regrettably, due to very common concomitant epilepsy, most girls with Rett syndrome cannot undergo the assessment of body composition using the method of electrical bioimpedance, which is a simple, accurate and non-invasive study evaluating parameters such as energy resources. Therefore, it may be important to measure the circumference of the arm and skinfolds and calculate their body composition, including energy resources, in assessing nutritional status. Arm circumference is a useful measure of body fat and lean mass, especially in children up to 5 years of age. It is particularly useful when body weight is not a reliable determinant of nutritional status (e.g., swelling, dehydration, chronic glucocorticoid therapy—GCS) or when a reliable measurement of body length/height cannot be obtained [16]. Energy resources of patients with Rett syndrome calculated on the basis of skinfolds were not large, but in most cases they were similar or within the normal limits commonly adopted for girls from the general population (>19%). The median of %FAT was lower than control group but the difference was not statistically significant, while the muscle mass and the muscle circumference of the arm were significantly lower in the study group than in the controls. However, there was a significant positive correlation with other anthropometric parameters, such as body weight, BMI z-score, waist circumference, hip circumference, %FAT and muscle mass. This confirms their usefulness in assessing the nutritional status in patients with Rett syndrome, especially in case of difficulties in performing some measurements using standard methods used in a healthy population.

According to Australian researchers who recruited an international panel of experts, analyzed the available literature and developed recommendations for parents and clinicians dealing with Rett syndrome, the assessment of energy requirements should be based on serial measurements of body height, but in case of underweight patients, energy requirement should exceed the recommended calorie intake for body weight [11]. Energy-dense foods are the best way to increase calorie intake. Snacks containing high-calorie products and high-calorie ONS can also be served [2,23,24]. Other authors also emphasized that most girls with this condition required crushing or mashing of products to make eating easier, which was confirmed by our research [7]. The diets of girls with Rett syndrome significantly more commonly included soft products which were easy to crush and perceived as providing a high energy value, i.e., bananas, potatoes or root vegetables in soups, as well as products providing a large amount of protein, i.e., cold cuts, cottage cheese and eggs. However, the diets of girls with Rett syndrome were characterized by a significantly lower energy value and carbohydrate content, including starch, sucrose and dietary fiber, compared to those in the control group. The diets of the majority of girls with Rett syndrome did not meet the nutritional standards for energy, iron and calcium. However, most of them complied with the norm for the intake of protein, sodium, potassium, phosphorus, magnesium, zinc and vitamins B_1_, B_2_, B_6_, B_12_, D. When the daily intake of nutrients was converted to kg of body weight (kg b.w), it was found that girls with Rett syndrome consumed statistically significantly more of each of the macro- and micronutrients analysed than the control group. This was mainly due to the more frequent use of supplements of vitamins and ONS. A low percentage of girls in the study group received a suitable amount of fluids. Similar data were reported by other authors. Most of the cohort studied by Chin et al. [22] had adequate protein and energy intake. Fiber intake was generally low in this group, and most individuals did not reach the daily reference intake. Protein intake was significantly lower in people with severe growth deficiency. Nevertheless, almost a third of the people participating in the study ate more than expected and less than a quarter ate less than expected. The low intake of fluids was a cause of concern. A study by Schwartzman et al. [2] showed no significant correlation between protein intake with diet and body height. Insufficient iron and calcium intake was observed. Motil et al. [6] found that the parents of the study participants reported that their children had a “good appetite”, although the total energy and calcium intake was significantly lower than the reference values for height and age.

Patients with Rett syndrome usually have a good appetite, and deficiencies in body weight and height are very often due to symptoms associated with the disease, such as holding breath, hyperventilation, hand stereotypies, upper body swaying, or feeding disorders requiring crushing, chopping or blending of food, and depend on the mobility of patients [11,21]. There is also prolonged feeding time, gagging or choking with food or liquids, and some patients require enteral nutrition support [7]. Poor feeding skills, including difficulty in chewing and swallowing food, and involuntary tongue movements, possibly due to altered muscle tone and dyspraxia, as well as poor motor control, excessive salivation, hyperventilation, and holding breath, may further interfere with feeding [7,25]. These difficulties are compounded by poor communication skills, which make it difficult for patients to express what or how much they want to eat. As a result, fewer calories may be consumed than are necessary for growth and development. One should also be aware of possible additional energy requirements associated with involuntary activities, including hand stereotypies and autonomic system abnormalities, including hyperventilation or breathing disorders. However, this has not been confirmed in clinical trials so far [11].

Thin liquids are the most challenging for patients with swallowing disorders because they flow quickly to the pharynx and require adequate lip closure, sensorimotor regulation and/or complete closure of the laryngeal vestibule [26]. Bianco and Rota [27] in their review indicated that children with RS present high-arched palate, oral breathing, sialorrhea, poor tongue movements, and open bites, which influence not only the oral phase of swallowing but also the pharyngeal. It is probable that common breathing abnormalities presented by RS girls may impact swallow safety [28]. Breathing–swallowing discoordination results from prolonged swallow latency, delayed timing of following sequences of swallowing, and breathing irregularities or hyperventilation [29]. Untreated malocclusion may also be associated with dysphagia severity in RS patients [30]. The described disorders may lead to an increased risk of aspiration (including silent without cough response), especially with thin liquids [31,32]. Presumably, insufficient fluid intake in our patients resulted from fluid avoidance by children or caregivers due to fear of choking or aspiration or extended efforts paid to fluids swallowing. However, little is known about the exact prevalence, severity, mechanism, and treatment of swallowing disorders in RETT syndrome patients [33].

Based on the reviewed literature, it seems that a diet containing the recommended daily intake of essential nutrients is optimal and should be given during frequent small feedings offered during the day. People at risk of the deficiency of certain micronutrients, e.g., those taking certain anticonvulsant drugs, poorly growing, with symptoms suggestive of malabsorption from the gastrointestinal tract or satisfying at least 50% of nutritional needs by enteral route, are recommended to undergo folic acid and vitamin B_12_ tests and possible supplementation should be considered [11].

Rett syndrome is a complex and overwhelming disorder. A multi-aspect medical treatment should be introduced using the knowledge of specialists working in multidisciplinary teams including a dietitian, because there is a lack of peer-reviewed literature with high scientific value in this area.

The limitation of our study was the inability to perform detailed body composition tests (e.g., BIA) due to comorbidities such as epilepsy. It would also be worth including the determination of markers of nutritional status in the blood and the saturation of the body with vitamins and minerals before a possible change in diet. The strength of our study was proving that the simple and cheap methods we used to assess nutritional status were good and useful and minimally invasive, especially if other methods, such as BIA, cannot be used because of epilepsy. The nutritional analysis of Polish girls with Rett syndrome is a groundbreaking study that will allow for better dietary planning and supplementation of this group of patients in order to prevent nutritional status disorders and other diet-related disorders, such as constipation and reflux, which are common in this group, which will improve the patients’ quality of life. It will also enable the development of national guidelines for nutritional management in this group of patients. This paper demonstrates the potential of outcomes research to provide a methodology for identifying what is needed to enable healthcare professionals to provide the right nutritional care for patients with Rett syndrome.

## 5. Conclusions

1. Girls with Rett syndrome have reduced weight, poor growth that deteriorates with age, and are at risk of nutritional deficiencies.
2. Various nutritional intervention strategies should be explored to reduce and, if possible, prevent malnutrition and cachexia in girls with Rett syndrome.
3. There is a need to systematically review the literature and gather expertise to identify current best practices in relation to nutritional assessment and management.

## Figures and Tables

**Table 1:** Indicators used for the food frequency questionnaire categories [18].

Consumption Frequency Categories	Ranks Assigned to Frequency Categories	Daily Frequency (Times/Day)
Never or almost never	1	0.0
Once a month or less frequently	2	0.025
Several times a month	3	0.100
Several times a week	4	0.571
Every day	5	1.000
Several times a day	6	2.000

**Table 2:** Measurements and anthropometric indicators.

Parameters	Study Group (*n* = 49)								Control Group (*n* = 22)								*p*
	Mean	SD	Q0 (Min)	Q1	Q2 (Median)	Q3	Q4 (Max)	IQR	Mean	SD	Q0 (Min)	Q1	Q2 (Median)	Q3	Q4 (Max)	IQR	
Age (years)	8.7	4.9	2.1	5.2	8.2	11.4	27.6	6.2	10.7	3.5	4.5	8.7	10.5	12.3	18.6	3.6	0.09
Body mass (kg)	22.3	11.4	9.4	15.7	19.4	26.4	57.5	10.7	38.4	13.5	16.3	27.9	38.6	47.0	73.5	19.0	**0.00**
Height (cm)	114.2	19.5	69.0	100.0	115.0	127.0	160.0	27.0	145.2	17.0	105.5	135.7	147.2	154.9	169.5	19.1	**0.00**
BMI (kg/m^2^)	16.7	6.9	9.8	12.8	14.8	17.2	52.9	4.3	17.7	3.4	13.9	15.1	16.9	19.1	27.0	3.9	0.15
BMI z-score	−0.8	2.2	−6.4	−2.3	−0.9	0.4	6.3	2.8	−0.1	1.1	−1.9	−0.7	−0.2	0.5	2.0	1.1	0.07
Waist circumference (cm)	56.3	10.5	39.0	49.0	54.0	65.0	78.0	16.0	63.4	14.9	47.0	55.2	60.0	64.7	118.0	9.5	**0.03**
Hip circumference (cm)	63.3	13.8	43.0	54.0	62.0	73.0	96.0	19.0	77.4	12.0	57.0	69.2	76.7	87.0	104.0	17.7	**0.00**
WHR	0.9	0.1	0.5	0.8	0.9	0.9	1.1	0.1	0.8	0.2	0.7	0.7	0.8	0.8	1.7	0.1	**0.00**
WHtR	0.5	0.1	0.3	0.4	0.5	0.5	1.1	0.1	0.4	0.1	0.4	0.4	0.4	0.4	1.0	0.0	**0.01**
Arm circumference (cm)	17.4	4.1	11.0	14.5	16.0	19.0	31.0	4.5	21.7	3.9	16.5	18.4	21.5	23.7	32.0	5.4	**0.00**
Muscle mass (kg)	13.7	2.9	7.0	11.8	13.2	15.8	23.1	3.9	17.1	3.0	12.7	15.5	16.3	18.0	25.1	2.5	**0.00**
Suprailiac skinfold * (mm)	8.5	7.5	2.0	4.0	6.0	12.0	45.0	8.0	12.0	12.9	4.0	6.2	10.0	12.0	67.0	5.7	0.06
Subscapular skinfold ** (mm)	8.5	4.9	3.0	5.0	7.0	11.0	24.0	6.0	9.5	5.5	5.0	5.2	8.5	10.0	26.0	4.7	0.38
Triceps skinfold *** (mm)	11.6	5.3	2.0	8.0	10.5	16.0	25.0	8.0	14.4	5.5	6.0	11.0	14.0	17.5	26.0	6.5	**0.04**
FAT (%)	22.9	6.7	10.0	17.6	20.9	28.3	36.0	10.7	25.6	5.7	15.6	21.8	24.3	28.8	36.5	6.9	0.09
Bioimpedance Analysis (BIA)																	
BMR (kcal/d)	828.0	99.6	701.0	733.0	843.0	866.0	1016.0	133.0	1326.2	205.8	887.0	1159.0	1375.0	1453.0	1685.0	294.0	**0.00**
FAT (%)	21.2	13.8	8.4	10.2	19.4	24.6	53.5	14.4	19.7	6.8	3.0	15.2	18.4	23.8	32.2	8.5	0.89
LBM (%)	79.4	14.0	46.5	75.4	83.1	89.8	91.6	14.4	79.6	6.8	67.8	75.5	80.9	83.2	96.9	7.7	0.44
TBW (l)	64.2	11.0	43.9	59.0	62.2	75.7	78.0	16.7	58.7	4.1	49.7	56.7	58.2	61.5	67.0	4.8	0.06
Cell mass (kg)	6.4	2.8	4.3	4.4	6.1	6.3	13.6	1.9	15.3	5.1	6.6	10.9	15.7	18.2	24.0	7.2	**0.00**
Muscle mass (kg)	3.8	0.9	2.8	2.9	4.3	4.4	4.6	1.6	12.1	4.2	4.7	8.4	12.1	13.8	19.9	5.4	**0.00**

BMI—body mass index, WHR—waist to hip ratio; WHtR—waist to height ratio; FAT—fatty adipose tissue; * Supra-iliac skinfold (above the upper bone of the hip); ** Subscapular skinfold (under the lowest point of the shoulder blade); *** Triceps skinfold (back side of the middle upper arm); FAT—fatty adipose tissue; BMR—basal metabolic rate; LBM—lean body mass; TBW—total body water.

**Table 3:** The number and percentage of patients, depending on the interpretation of BMI.

	Study Group *n* (%)	Control Group *n* (%)
Significantly underweight	19 (38.78)	0
Underweight	5 (10.2)	3 (13.64)
Normal	18 (36.73)	16 (72.73)
Overweight	2 (4.08)	2 (9.09)
Obese	3 (6.12)	1 (4.55)
Extremely obese	2 (4.08)	0 (0.0)
Total	49 (100)	22 (100)

**Table 4:** Data from the food frequency questionnaire.

Group of Products	Average Times Per Day		*p*
	Study Group	Control Group	
1. Sugar for sweetening drinks	0.56	0.30	0.27
2. Honey for sweetening drinks	0.41	0.23	0.35
3. Chocolates (chocolate candies and chocolate bars)	0.10	0.39	**0.00**
4. Non-chocolate candies (e.g., fruit candies, caramels, gummies, fudge, toffees)	0.03	0.15	**0.00**
5. Biscuits and cookies	0.14	0.23	**0.01**
6. Ice cream and pudding	0.07	0.21	**0.00**
7. Salty snacks	0.09	0.18	**0.00**
8. Milk and natural milk drinks	0.56	0.68	0.40
9. Sweetened milk drinks	0.34	0.26	0.99
10. Cocoa	0.07	0.18	0.07
11. Natural cottage cheeses	0.18	0.25	0.62
12. Cottage cheese with flavor additives	0.12	0.04	0.09
13. Cheese (yellow)	0.29	0.46	0.69
14. Other cheeses	0.08	0.09	0.92
15. Eggs and egg dishes where eggs are the main ingredient of the dish	0.42	0.26	0.07
16. Wholegrain bread or bread with grains (so-called dark)	0.48	0.46	0.39
17. Refined bread (so-called white)	0.64	0.58	0.90
18. Unrefined coarse grains, brown rice, wholegrain pasta	0.18	0.22	0.67
19. Refined fine grain groats	0.16	0.09	0.42
20. White rice	0.15	0.21	0.66
21. Pasta	0.22	0.22	0.85
22. Breakfast cereals	0.24	0.24	0.26
23. Canola oil	0.37	0.46	0.17
24. Soybean oil	0.01	0.00	1.00
25. Flaxseed oil	0.08	0.01	0.39
26. Olive oil	0.20	0.27	0.11
27. Other vegetable oils	0.09	0.02	0.33
28. Butter	1.01	0.71	0.13
29. Margarine cubes	0.06	0.01	0.81
30. Margarine in cups	0.07	0.03	0.32
31. Cream	0.28	0.22	0.82
32. Other animal fats	0.02	0.01	0.15
33. Mayonnaise and dressings	0.07	0.02	0.18
34. Fruits	0.88	0.94	0.67
35. Stone fruits	0.25	0.36	0.32
36. Kiwi and citrus	0.21	0.43	**0.02**
37. Other tropical fruits	0.13	0.24	0.13
38. Berry fruit	0.21	0.41	0.10
39. Bananas	0.49	0.26	**0.01**
40. Apples and pears	0.45	0.52	0.32
41. Avocado	0.08	0.12	0.92
42. Olives	0.03	0.15	**0.02**
43. Dried fruits	0.11	0.06	0.57
44. Sweet fruit preserves	0.19	0.12	0.31
45. Vegetables	1.02	0.89	0.2
46. Cruciferous vegetables	0.28	0.29	0.76
47. Yellow-orange vegetables	0.62	0.47	0.21
48. Leafy green vegetables	0.20	0.27	0.83
49. Tomatoes	0.43	0.52	0.43
50. Vegetables such as cucumber	0.34	0.51	**0.04**
51. Root and other vegetables	0.48	0.34	**0.04**
52. Fresh legumes and canned seeds	0.13	0.22	0.48
53. Dry legume seeds	0.03	0.13	0.94
54. Soy	0.00	0.11	0.55
55. Dry legumes in dishes	0.03	0.14	0.75
56. Potatoes	0.56	0.38	**0.04**
57. Walnuts	0.07	0.08	0.12
58. Peanuts	0.02	0.04	0.38
59. Other nuts	0.05	0.14	**0.00**
60. Nut creams	0.05	0.06	0.16
61. Seeds	0.06	0.08	0.40
62. Flax (Flax seeds)	0.06	0.03	**0.02**
63. Sausages	0.32	0.16	0.12
64. High-quality sausage (cold cuts)	0.56	0.35	0.20
65. Sausage products and offal	0.10	0.08	0.06
66. Red meat	0.24	0.19	0.33
67. Poultry and rabbit meat	0.42	0.35	0.26
68. Venison	0.01	0.06	0.71
69. Lean fish	0.08	0.09	0.88
70. Fatty fish	0.06	0.08	0.48
71. Fruit juices and fruit nectars	0.28	0.45	0.08
72. Vegetable and vegetable-fruit juices	0.08	0.18	0.56
73. Energy drinks	0.00	0.09	0.56
74. Sweetened drinks	0.00	0.04	**0.00**

**Table 5:** Dietary intake (7-day average).

Nutrients (Daily Intake)	Study Group (*n* = 49)								Control Group (*n* = 22)								*p*
	Mean	SD	Q0 (Min)	Q1	Q2 (Median)	Q3	Q4 (Max)	IQR	Mean	SD	Q0 (Min)	Q1	Q2 (Median)	Q3	Q4 (Max)	IQR	
Energy (kcal)	1352.5	476.4	615.4	1101.8	1248.5	1572.1	3577.0	470.3	1611.8	361.8	1019.9	1398.2	1683.9	1778.6	2218.4	380.4	**0.04**
(%EAR)	87.6	26.6	43.9	70.1	80.1	101.5	160.3	31.4	88.2	26.3	50.9	66.2	84.7	106.3	143.1	40.1	0.91
(kcal/kg b.w)	70.1	28.8	25.4	53.1	63.3	90.8	135.5	37.7	47.0	19.1	14.8	33.7	46.4	54.6	96.6	20.9	**0.00**
Protein (g)	56.3	23.2	26.6	42.8	52.6	67.3	176.2	24.5	60.6	16.7	36.2	46.0	60.9	70.1	93.2	24.0	0.19
(%RDA)	213.8	106.6	81.7	130.1	183.9	257.2	513.6	127.1	172.3	76.6	72.0	107.6	153.5	221.5	348.6	113.9	0.10
(g/kg b.w)	2.9	1.3	0.9	2.1	2.5	3.8	6.7	1.8	1.8	0.8	0.5	1.0	1.7	2.3	3.6	1.2	**0.00**
Fats (g)	44.5	20.8	16.5	31.5	41.6	51.1	141.2	19.6	44.9	14.1	23.2	34.5	45.5	51.8	75.4	17.3	0.47
(%RDA)	82.3	28.0	29.5	64.6	79.9	95.7	163.1	31.1	72.7	24.2	37.5	59.5	72.1	89.1	145.0	29.6	0.12
(g/kg b.w)	2.3	1.0	0.6	1.6	2.0	2.8	5.3	1.1	1.3	0.4	0.4	1.1	1.2	1.6	2.1	0.5	**0.00**
Carbohydrates (g)	188.3	67.2	88.9	137.7	181.6	212.4	411.7	74.7	249.7	60.5	128.3	198.1	257.0	295.5	353.5	97.5	**0.00**
(g/kg b.w)	9.8	4.3	3.3	7.1	8.9	12.3	20.1	5.3	7.4	3.4	2.2	4.8	6.9	8.5	16.3	3.6	**0.02**
**Amino acids**																	
Isoleucine (mg)	2532.9	11,422	64.7	1917.0	2366.6	2868.8	8095.3	951.8	2810.1	829.8	1644.0	2138.5	2806.2	3298.4	4521.3	1159.9	0.13
Leucine (mg)	4066.9	1900.3	111.3	3078.7	3823.2	4706.9	13,629.4	1628.2	4630.2	1397.1	2578.9	3636.3	4717.0	5230.2	7398.5	1593.9	0.07
Lysine (mg)	3588.6	1662.7	61.8	2609.9	3308.3	4273.7	11,444.2	1663.8	3807.3	1139.5	1987.7	2862.3	3829.4	4518.0	6453.6	1 65.7	0.23
Methionine (mg)	1264.1	562.4	36.9	966.5	1189.2	1437.3	3982.5	470.8	1402.4	421.4	746.1	1124.6	1423.4	1650.3	2234.7	525.7	0.12
Cystine (mg)	731.5	275.8	43.8	602.1	701.6	799.1	1894.9	197.0	933.7	267.8	496.9	741.2	930.6	1117.8	1508.8	376.6	**0.00**
Phenylalanine (mg)	2299.7	1047.3	76.96	1819.5	2148.9	2663.9	7588.0	844.5	2646.7	763.6	1554.8	2029.3	2708.8	3009.2	4115.9	979.9	0.06
Tryptophan (mg)	670.2	311.5	15.9	510.5	638.8	773.5	2236.4	262.9	746.6	218.0	443.0	547.4	733.3	868.5	1258.4	321.1	0.09
**Minerals**																	
Sodium (mg)	2275.7	1021.4	773.2	1661.8	2074.4	2658.6	6555.1	996.8	2500.8	629.2	1439.0	2049.9	2585.1	2812.8	3731.2	762.9	0.08
(%AI)	201.3	73.6	68.3	142.7	197.2	239.2	437.0	96.5	196.6	57.8	95.9	170.0	191.1	248.1	286.2	78.1	0.90
(mg/kg b.w)	115.5	51.8	29.0	86.0	107.5	134.9	248.4	48.9	74.9	34.9	20.3	47.8	79.1	94.4	163.5	46.6	**0.00**
Potassium (mg)	2198.9	894.0	957.1	1625.8	1983.4	2496.8	5596.0	871.0	2250.8	611.2	1337.4	1800.8	2235.1	2552.3	4106.9	751.7	0.29
(%AI)	146.5	74.5	48.9	91.9	124.2	189.9	383.7	98.0	110.2	52.4	43.4	78.5	90.6	138.7	232.7	60.2	**0.03**
(mg/kg b.w)	112.9	50.5	44.8	71.9	105.7	140.2	254.2	68.3	66.2	29.6	22.1	50.0	57.2	75.5	129.9	25.5	**0.00**
Calcium (mg)	581.8	299.5	175.5	366.7	519.2	731.6	1773.0	364.9	596.3	218.8	266.1	457.1	611.4	681.9	1210.0	224.8	0.59
(%RDA)	58.5	27.4	17.6	33.6	58.4	74.3	136.4	40.8	51.7	21.4	20.5	36.8	47.6	65.6	121.0	28.8	0.35
(mg/kg b.w)	30.4	17.5	6.6	19.1	26.9	37.9	87.4	18.9	17.6	8.8	4.6	9.9	17.3	22.1	40.9	12.2	**0.00**
Phosphorus (mg)	870.2	359.5	398.9	664.3	813.9	1002.2	2649.5	337.9	960.1	240.8	558.2	780.4	967.1	1115.7	1391.3	335.3	0.06
(%RDA)	134.7	53.5	51.7	91.7	121.9	176.5	304.1	84.8	115.9	58.9	50.0	69.3	93.0	159.1	231.9	89.8	0.12
(mg/kg b.w)	45.6	21.8	12.9	29.6	38.4	59.6	100.4	30.0	28.3	12.5	9.1	16.4	29.0	34.4	58.9	17.9	**0.00**
Magnesium (mg)	204.0	80.3	88.6	147.6	185.3	237.3	525.8	89.7	226.2	47.3	152.9	193.6	228.7	255.9	356.4	62.3	**0.04**
(%RDA)	138.9	67.0	35.7	81.1	128.2	177.8	275.8	96.8	113.6	59.5	54.1	68.2	83.1	148.5	274.1	80.3	0.13
(mg/kg b.w)	10.5	4.8	3.8	6.9	9.4	13.6	22.5	6.6	6.6	2.5	2.6	4.9	6.6	7.4	12.1	2.4	**0.00**
Iron (mg)	9.4	5.9	3.5	6.0	8.2	10.5	39.9	4.5	9.3	3.6	6.0	6.9	8.5	9.6	21.6	2.7	0.54
(%RDA)	92.8	57.5	35.0	61.9	83.2	106.5	398.6	44.6	78.8	29.5	43.3	62.6	69.3	88.8	146.4	26.2	0.35
(mg/kg b.w)	0.5	0.3	0.2	0.3	0.4	0.5	2.3	0.3	0.3	0.1	0.1	0.2	0.2	0.3	0.5	0.2	**0.00**
Zinc (mg)	7.3	3.6	3.4	4.9	6.3	7.9	22.6	2.9	6.8	1.4	4.2	5.8	6.9	7.4	10.2	1.6	0.55
(%RDA)	135.8	66.4	55.0	95.4	120.4	166.7	459.5	71.4	101.3	43.6	52.1	69.1	84.0	137.	203.8	68.3	**0.01**
(mg/kg b.w)	0.4	0.2	0.1	0.2	0.3	0.5	1.4	0.2	0.2	0.1	0.1	0.1	0.2	0.2	0.4	0.1	**0.00**
Copper (mg)	0.8	0.5	0.4	0.6	0.7	0.9	2.8	0.3	0.9	0.3	0.5	0.8	0.9	1.15	1.6	0.4	**0.01**
(%RDA)	147.9	66.3	54.8	96.6	124.5	183.3	311.2	86.6	137.9	44.8	74.3	110.1	129.3	160.1	243.6	50.0	0.97
(mg/kg b.w)	0.04	0.02	0.02	0.03	0.04	0.05	0.09	0.02	0.03	0.01	0.01	0.02	0.03	0.03	0.05	0.01	**0.00**
Manganese (mg)	3.1	1.4	0.9	2.3	2.9	3.5	7.0	1.3	2.8	0.8	1.5	2.3	2.6	3.2	4.4	1.0	0.46
(%AI)	197.4	74.7	77.6	151.5	191.2	232.5	391.7	80.9	171.1	56.4	92.5	129.0	154.6	204.8	293.8	75.8	0.16
(mg/kg b.w)	0.1	0.1	0.1	0.1	0.1	0.2	0.3	0.1	0.1	0.04	0.02	0.1	0.1	0.1	0.2	0.04	**0.00**
**Vitamins**																	
Retinol (ug)	1171.4	4362.1	61.9	198.1	267.9	485.2	30,013.1	287.0	240.4	108.5	101.9	164.0	210.3	267.7	512.8	103.7	0.06
(ug/kg b.w)	46.1	127.3	4.3	9.4	13.9	25.7	850.2	16.3	7.1	3.9	1.8	3.8	6.7	10.2	14.7	6.4	**0.00**
B_1_ (mg)	2.3	7.3	0.3	0.7	0.9	1.4	52.4	0.7	0.9	0.3	0.4	0.7	0.8	0.9	1.7	0.2	0.17
(%RDA)	258.7	668.0	52.9	90.2	111.1	213.4	4759.9	123.3	93.3	33.3	38.3	67.7	81.4	113.2	153.3	45.6	**0.00**
(mg/kg b.w)	0.1	0.2	0.02	0.03	0.05	0.1	1.5	0.05	0.02	0.01	0.01	0.02	0.02	0.03	0.05	0.01	**0.00**
B_2_ (mg)	2.7	7.3	0.5	1.1	1.4	1.9	52.5	0.9	1.4	0.5	0.7	0.9	1.3	1.6	3.0	0.6	0.26
(%RDA)	310.2	661.8	98.0	121.8	173.5	264.1	4771.2	142.3	145.8	57.8	65.4	101.0	127.8	186.3	275.3	85.3	**0.01**
(mg/kg b.w)	0.1	0.2	0.02	0.05	0.1	0.1	1.5	0.1	0.04	0.02	0.01	0.02	0.04	0.1	0.1	0.04	**0.00**
Niacin (mg)	22.6	73.9	3.6	8.5	11.7	13.9	528.1	5.5	13.7	6.1	5.2	9.7	12.5	15.2	33.2	5.5	0.26
(%RDA)	199.1	524.6	35.2	87.1	109.7	137.5	3771.9	50.3	115.3	53.6	37.4	83.5	101.7	130.2	261.3	46.7	0.55
(mg/kg b.w)	0.9	2.1	0.1	0.4	0.5	0.7	14.9	0.3	0.4	0.3	0.11	0.2	0.4	0.5	1.0	0.3	**0.00**
C (mg)	275.4	785.6	21.2	38.0	63.9	135.1	5225.0	97.0	85.9	61.3	8.4	42.3	77.8	111.4	299.5	69.2	0.98
(%RDA)	494.3	1261.3	42.4	76.1	141.2	262.0	8038.5	185.9	155.4	123.5	16.9	83.7	130.9	169.5	598.9	85.8	0.63
(mg/kg b.w)	12.9	29.2	0.9	2.1	3.16	6.3	148.0	4.2	2.5	2.2	0.2	1.2	1.9	3.2	9.5	2.0	**0.00**
A (ug)	1826.8	4472.3	271.7	606.9	793.1	1268.8	31,278.2	661.8	946.9	681.7	195.1	440.9	764.1	1154.6	2818.9	713.7	0.18
(%RDA)	327.1	643.8	54.3	120.8	166.3	298.5	4468.3	177.8	171.5	119.5	32.5	68.0	149.9	265.5	469.8	197.5	0.12
(ug/kg b.w)	81.8	132.4	12.2	30.3	39.9	80.8	886.1	50.5	28.3	23.0	4.8	10.6	21.8	38.5	92.9	27.8	**0.00**
E (mg)	35.5	111.4	2.2	3.8	6.1	12.8	753.2	8.9	6.0	2.5	2.5	4.6	5.5	7.1	12.1	2.4	0.58
(%AI)	503.9	1594.2	34.2	56.5	83.6	154.8	10,760.5	98.3	79.0	35.6	31.2	55.8	73.5	95.1	170.5	39.3	0.22
(mg/kg b.w)	1.9	0.3	0.1	0.2	0.3	0.5	29.5	0.3	0.2	0.1	0.03	0.1	0.2	0.2	0.4	0.1	**0.00**
B_6_ (mg)	2.8	7.6	0.6	1.02	1.5	2.1	54.7	1.0	1.4	0.5	0.8	1.1	1.3	1.5	3.2	0.4	0.36
(%RDA)	311.5	646.8	72.1	130.3	170.5	260.5	4558.8	130.2	142.1	60.1	67.5	102.1	122.2	162.2	279.8	60.1	**0.01**
(mg/kg b.w)	0.1	0.2	0.02	0.05	0.1	0.1	1.5	0.05	0.04	0.02	0.01	0.03	0.04	0.05	0.1	0.02	**0.00**
B_12_ (ug)	8.4	36.5	0.5	1.9	2.4	2.9	256.4	1.0	2.2	1.0	1.0	1.5	1.9	2.7	5.3	1.2	0.10
(%RDA)	429.7	1514.4	30.1	96.9	144.9	265.2	10,684.5	168.3	122.6	58.9	41.2	74.2	114.9	169.3	222.9	95.1	**0.02**
(ug/kg b.w)	0.3	1.0	0.03	0.1	0.1	0.2	7.3	0.1	0.1	0.04	0.02	0.04	0.05	0.09	0.2	0.05	**0.00**
D (ug)	47.5	86.1	0.5	12.9	28.0	51.6	511.1	38.7	2.1	1.8	0.6	1.2	1.8	2.09	9.4	0.9	**0.00**
(%RDA)	316.6	574.1	3.0	86.1	186.8	344.3	3407.3	258.2	14.0	12.3	3.8	8.1	12.4	13.9	62.5	5.9	**0.00**
(ug/kg b.w)	2.3	3.1	0.02	0.9	1.6	2.6	16.2	1.9	0.1	0.05	0.01	0.04	0.05	0.1	0.2	0.03	**0.00**
Folate (ug)	247.6	163.8	96.6	152.9	203.8	265.1	971.7	112.1	218.3	91.3	115.7	161.1	190.5	244.1	452.1	83.0	0.72
(%RDA)	95.9	54.1	37.2	63.5	76.9	116.3	248.6	52.8	69.9	29.2	28.9	46.8	63.3	80.2	150.7	33.4	**0.05**
(ug/kg b.w)	12.4	7.4	3.9	7.4	9.8	14.6	36.8	7.2	6.3	3.1	1.6	4.5	5.9	8.1	14.3	3.6	**0.00**
**Other**																	
Glucose (g)	7.5	5.2	1.1	3.9	5.8	9.4	23.9	5.4	7.3	4.7	0.8	4.5	6.6	8.8	22.4	4.3	0.89
Saccharose (g)	44.1	25.3	4.5	28.7	39.2	51.8	139.8	23.0	50.8	16.8	17.1	41.3	52.4	61.2	93.6	19.9	**0.04**
(g/kg b.w)	2.3	1.4	0.1	1.4	2.2	2.9	6.2	1.5	1.5	0.8	0.4	1.0	1.2	2.0	3.2	1.0	**0.02**
Fructose (g)	9.8	6.8	0.9	4.8	8.2	12.9	28.7	8.1	10.9	8.1	0.7	5.9	9.3	14.3	36.6	8.4	0.57
Lactose (g)	8.4	7.9	0.2	2.7	6.0	10.3	32.4	7.6	8.9	5.5	0.6	4.9	7.7	12.9	21.3	7.9	0.40
Starch (g)	75.6	35.6	6.6	58.3	72.1	91.8	189.5	33.5	125.0	37.5	48.9	102.5	132.3	139.0	213.7	36.5	**0.00**
Dietary fiber (g)	13.2	5.6	5.4	9.8	11.7	16.1	29.3	6.2	16.7	4.9	8.6	13.5	16.4	20.8	26.8	7.3	**0.00**
Dietary fiber (%AI)	86.9	32.8	34.6	63.9	84.6	103.4	183.2	39.5	96.6	31.6	47.3	72.5	107.1	112.9	167.5	40.5	0.20
(g/kg b.w)	0.7	0.3	0.2	0.4	0.6	0.8	1.7	0.3	0.5	0.2	0.1	0.3	0.4	0.6	1.0	0.2	**0.01**
Cholesterol (mg)	234.8	135.0	19.7	155.2	209.9	277.2	749.8	121.9	196.6	84.5	64.2	138.8	177.1	264.1	374.5	125.3	0.33
(mg/kg b.w)	12.8	8.9	0.6	6.9	9.6	17.6	42.6	10.7	5.8	3.3	1.6	3.2	5.1	7.9	12.6	4.7	**0.00**
SFA (g)	18.9	11.4	6.4	12.3	15.7	23.1	63.4	10.8	17.3	7.3	8.1	12.1	15.3	21.2	36.3	9.1	0.73
SFA (%AI)	132.4	81.9	40.5	85.4	116.9	140.6	506.9	55.3	120.5	52.4	64.6	83.6	99.3	143.4	283.9	59.8	0.81
(g/kg b.w)	0.9	0.5	0.2	0.6	0.9	1.2	2.6	0.5	0.5	0.2	0.1	0.4	0.5	0.7	1.0	0.3	**0.00**
MUFA (g)	14.5	7.6	0.8	10.2	13.8	18.9	50.7	8.8	16.7	5.7	6.8	12.2	16.4	19.9	27.9	7.7	0.11
18:2	3.8	2.2	0.2	2.5	3.2	4.6	13.2	2.1	5.8	2.7	2.5	3.3	5.5	7.5	10.7	4.1	**0.00**
18:3	0.8	0.5	0.1	0.4	0.6	0.8	2.6	0.4	0.9	0.6	0.4	0.5	0.7	1.3	2.5	0.8	0.16
PUFA (g)	5.1	2.7	1.4	3.2	4.3	6.7	14.9	3.5	7.2	3.3	3.6	4.3	6.4	9.1	12.8	4.7	**0.00**
n-3	0.9	0.8	0.2	0.5	0.8	1.2	4.6	0.7	1.2	0.8	0.4	0.6	0.9	1.6	3.5	1.0	0.20
n-6	3.9	2.2	0.2	2.5	3.3	4.7	13.2	2.2	5.9	2.7	2.6	3.4	5.5	7.5	10.7	4.1	**0.00**
Salt (g)	5.6	2.6	1.9	4.2	5.1	6.5	16.4	2.3	6.0	1.6	3.6	4.9	5.9	7.0	9.3	2.2	0.15
Water (mL)	1361.4	499.6	624.8	999.2	1243.7	1550.8	3129.2	551.7	1434.8	520.2	599.9	1069.4	1383.8	1866.7	2359.5	797.2	0.48
(%AI)	81.7	26.6	39.0	64.5	75.8	95.5	164.7	30.9	78.5	28.9	31.8	56.5	74.9	105.4	121.0	48.9	0.83
(ml/kg b.w)	69.9	28.5	26.4	50.6	62.4	93.5	134.9	42.9	42.0	20.2	13.2	21.8	44.6	56.6	92.0	34.8	**0.00**
%E from P	16.7	2.6	10.3	15.1	16.6	18.6	22.1	3.5	15.0	2.0	10.8	13.4	15.6	16.2	18.4	2.8	**0.02**
%E from F	29.3	6.7	11.9	24.4	28.4	34.5	50.2	10.2	24.9	5.6	12.9	21.7	24.7	28.6	34.8	6.9	**0.01**
%E from C	52.1	7.2	33.7	46.7	51.4	56.9	73.4	10.2	58.0	5.4	47.3	54.2	57.9	61.1	71.7	6.9	**0.00**

RDA—Recommended Dietary Allowance; AI—Adequate Intake; EAR—Estimated Average Requirement; SFA—Saturated Fatty Acids; MUFA—Monounsaturated Fatty Acids; PUFA—Polyunsaturated Fatty Acids; %E from P—% of energy contribution from proteins; %E from F—% of energy contribution from fats; %E from C—% of energy contribution from carbohydrates, b.w—body weight.

**Table 6:** Number and percentage of respondents whose diets met the reference norms for the selected nutrient.

Nutrient [Type of Norms]	Study Group (Implementation of the Norm)		Control Group (Implementation of the Norm)	
	*n*	%	*n*	%
Energy (%EAR)	13	26.5	7	31.8
Protein (%RDA)	46	93.9	15	68.2
Fat (%RDA)	9	18.4	1	4.5
Sodium (%AI)	48	97.9	21	95.4
Potassium (%AI)	36	73.5	7	31.8
Calcium (%RDA)	3	6.1	1	4.5
Phosphosus (%RDA)	35	71.4	10	45.4
Magnesium (%RDA)	33	67.3	10	45.4
Iron (%RDA)	15	30.6	4	18.2
Zinc (%RDA)	30	61.2	8	36.4
Copper (%RDA)	35	71.4	17	77.3
Manganese (%AI)	44	89.8	20	90.9
B_1_ (%RDA)	32	65.3	9	40.9
B_2_ (%RDA)	49	100.0	16	72.7
Niacin (%RDA)	30	61.2	13	59.1
C (%RDA)	31	63.3	14	63.6
A (%RDA)	47	95.9	13	59.1
E (%AI)	20	40.8	13	59.1
B_6_ (%RDA)	42	85.7	17	77.3
B_12_ (%RDA)	35	71.4	11	50.0
D (%RDA)	35	71.4	0	0.0
Folates (%RDA)	16	32.6	3	13.6
Dietary fiber (%AI)	17	34.7	11	50.0
SFA (%AI)	32	65.3	11	50.0
Water (%AI)	9	18.4	7	31.8

**Table 7:** Spearman’s rank order correlation for the study group.

Variables	Study Group						
	N (Important)	BMI z-Score		Arm Circumference		Muscle Mass	
		R (Spearman)	*p*	R (Spearman)	*p*	R (Spearman)	*p*
Body mass	49	0.57	**0.00**	0.82	**0.00**	0.76	**0.00**
BMI z-score	49	-	**-**	0.69	**0.00**	0.53	**0.00**
Waist circumference	49	0.67	**0.00**	0.79	**0.00**	0.72	**0.00**
Hip circumference	49	0.69	**0.00**	0.85	**0.00**	0.76	**0.00**
Arm circumference	49	0.69	**0.00**	-	**-**	0.92	**0.00**
Muscle mass	49	0.53	**0.00**	0.92	**0.00**	-	**-**
Suprailiac skinfold	49	0.66	**0.00**	0.63	**0.00**	0.59	**0.00**
Subscapular skinfold	49	0.71	**0.00**	0.77	**0.00**	0.70	**0.00**
Triceps skinfold	49	0.79	**0.00**	0.73	**0.00**	0.46	**0.00**
% FAT	49	0.79	**0.00**	0.79	**0.00**	0.61	**0.00**

FAT—fatty adipose tissue.

**Table 8:** Spearman’s rank order correlation for the control group.

Variables	Control Study						
	N (Important)	BMI z-Score		Arm Circumference		Muscle Mass	
		R (Spearman)	*p*	R (Spearman)	*p*	R (Spearman)	*p*
Body mass	22	0.36	0.09	0.89	**0.00**	0.88	**0.00**
BMI z-score	22	-	-	0.63	**0.00**	0.32	0.14
Waist circumference	22	0.63	**0.00**	0.90	**0.00**	0.79	**0.00**
Hip circumference	22	0.33	0.12	0.87	**0.00**	0.88	**0.00**
Arm circumference	22	0.63	**0.00**	-	-	0.89	**0.00**
Muscle mass	22	0.32	0.14	0.89	**0.00**	-	-
Suprailiac skinfold	22	0.44	**0.04**	0.36	0.10	0.26	0.24
Subscapular skinfold	22	0.71	**0.00**	0.74	**0.00**	0.61	**0.00**
Triceps skinfold	22	0.85	**0.00**	0.67	**0.00**	0.32	0.14
% FAT	22	0.87	**0.00**	0.75	**0.00**	0.48	**0.02**

FAT—fatty adipose tissue.

## Data Availability

Data will be released upon the request of the data subject. The data are archived in the database of the Medical University of Warsaw.
